# Maternal knowledge and attitude towards unintentional childhood injury among children under five

**DOI:** 10.4102/ajod.v14i0.1617

**Published:** 2025-05-15

**Authors:** Olaitan J. Balogun, Oyeronke O. Bello, Loveness A. Nkhata, Joseph Conran

**Affiliations:** 1 Department of Health and Rehabilitation Sciences, Physiotherapy Division, Stellenbosch University, Cape Town, South Africa; 2 Division of Pediatrics, College of Medicine, University of Ibadan, Ibadan, Nigeria

**Keywords:** unintentional injury, maternal, knowledge, attitude, under-fives, nature of injuries

## Abstract

**Background:**

Childhood injuries resulting in disability represent a critical global health challenge, particularly for children under five and their families. Unintentional injuries, including falls, fractures, burns, scalds, and poisoning, pose significant risks. In Oyo State, Nigeria, limited maternal knowledge about these injuries potentially contributes to inadequate prevention strategies.

**Objectives:**

The study examined unintentional childhood injuries among children under five by identifying nature of injury, assessing maternal knowledge and attitudes, and exploring associations between socio-demographic factors and their knowledge and attitude.

**Method:**

A cross-sectional survey was conducted across two hospitals, employing a structured questionnaire to collect data on injury characteristics, maternal knowledge, and attitudes. Statistical analysis using SPSS version 23.0 involved percentage calculations, standard deviation, Fisher’s exact test and chi-square test to evaluate demographic variable associations at a 5% significance level.

**Results:**

Findings revealed falls, scalds, soft tissue damage, poisoning, and burns as the most frequent unintentional injuries necessitating hospitalisation. The research uncovered a significant deficit in maternal knowledge about childhood injuries, accompanied by predominantly negative preventive attitudes. Mothers’ age, religious background, and educational attainment demonstrated statistically significant correlations with knowledge scores.

**Conclusion:**

The study exposes critical gaps in understanding and preventing childhood injuries, emphasising the urgent need for targeted educational interventions across community stakeholders to mitigate risks and improve child health outcomes.

**Contribution:**

The study contributes to the existing literature, identified specific knowledge deficits regarding childhood injury prevention and factors that influence preventive knowledge and attitude. It also provides an evidence-base for developing appropriate educational interventions targeting vulnerable population.

## Introduction

Childhood injuries are an escalating global health concern, particularly affecting children from the age of one, contributing significantly to disability and mortality rates (WHO & UNICEF 2005). Unintentional injuries encompass a range of incidents, with falls resulting in fractures and trauma being the most prevalent, followed by road traffic accidents, drowning, poisoning and burns (Jullien 2021). Among children under five, these injuries pose a particular concern, with the unintentional injury mortality rate reaching 73 per 100 000, resulting in 3654 years of life lost per 100 000 population.

Falls resulting in fractures and trauma represent the most common form of unintentional injury in this age group, accounting for approximately 40% of all emergency department visits for children under five (World Health Organization [WHO] 2020). The risk of severe injury from falls is particularly high during developmental stages when children are learning to walk and climb, with the potential for long-term disabilities including traumatic brain injuries and permanent physical impairments.

These injuries can be intentional or unintentional, with the latter including incidents such as road traffic accidents, drowning, poisoning, burns and falls (Jullien 2021). In children under five, the unintentional injury mortality rate is alarmingly high, at 73 per 100 000, resulting in a loss of 3654 years of life per 100 000 population. In industrialised nations, these injuries are the leading cause of death and disability for this age group (WHO 2015). The WHO reports that child injury death rates are 3.4 times higher in low- and middle-income countries (LMICs) compared to high-income countries, with significant variations based on injury type.

Unintentional injuries are typically classified into categories: poisoning, burns, drowning, falls and transport-related incidents (WHO 2020). These injuries occur because of excessive exposure to various forms of energy, including mechanical, thermal, electrical, chemical or radiant (Centers for Disease Control and Prevention [CDC] 2012). They are often predictable and preventable through proper safety measures (Global Burden of Diseases 2020). Children’s natural curiosity and lack of awareness of risks make them particularly vulnerable to injuries, which place a significant burden on families and communities and can hinder development. Parents and caregivers play a crucial role in preventing these injuries, but a lack of knowledge often heightens the risk, contributing to rising mortality rates among children under five (Jullien 2021). Common accidents include vehicle-related incidents, poisoning, falls, thermal injuries and drowning. Despite improvements in child health care leading to a decline in infectious disease-related mortality (Mortality and Causes of Death Collaborators 2015), unintentional injuries remain a critical concern.

In Nigeria, poisoning is a significant cause of morbidity and mortality among children, often resulting from the ingestion of harmful substances. It accounts for 10% of unintentional injuries in children in LMICs, with 15% occurring in those under five, though many cases go unreported (Kavinda Chandimal Dayasiri, Jayamanne & Jayasinghe 2017). Factors contributing to childhood poisoning include children’s impulsiveness and curiosity, as well as environmental risks such as proximity to harmful substances. Addressing childhood injuries is essential for achieving the Sustainable Development Goal (SDG 3) related to good health and well-being. This includes enhancing parental education and awareness regarding injury prevention strategies (United Nations Bulletin 2023). Poor supervision and a lack of knowledge among mothers significantly increase the risk of unintentional injuries, highlighting the need for improved education and practices (Siu et al. 2019).

Research indicates that maternal education, age and knowledge are critical factors influencing childhood injury prevention (Inbaraj et al. 2020). In addition, the number of siblings, or parity, can affect the level of supervision children receive, though it does not significantly correlate with the incidence of home accidents (Shinde, Partel & Chavan 2022). This study aims to explore maternal knowledge and attitudes towards unintentional childhood injuries among children under five in Ogbomoso, Oyo State, Nigeria. The objectives include identifying common types of unintentional injuries, assessing maternal knowledge and attitudes regarding these injuries, and examining the association between socio-demographic characteristics and respondents’ knowledge scores.

### Theoretical framework

Protection Motivation Theory (PMT), developed by Rogers in 1975 and revised in 1983, explains how persuasive communication influences behaviour by focussing on the cognitive mechanisms that determine whether individuals adopt recommended protective behaviours.

In the context of this study, PMT provides a framework for understanding how mothers’ perceptions of injury risks and their ability to prevent them influence their protective behaviours. The theory’s key components directly inform our research objectives. Perceived severity of threat refers to mothers’ understanding of the consequences of injuries, while perceived vulnerability relates to their assessment of the likelihood of such injuries occurring. Response efficacy reflects their belief in the effectiveness of preventive measures, and self-efficacy represents their confidence in their ability to implement these strategies successfully. This theoretical framework guided our methodology, particularly in developing survey instruments that assess both knowledge (threat appraisal) and attitudes (coping appraisal) regarding unintentional childhood injuries.

Protection Motivation Theory evolved from the Health Belief Model that Rogers initially proposed and serves as a framework for understanding health behaviour change in response to perceived risks. According to PMT, individuals are more likely to engage in protective behaviours when faced with threats. The theory emphasises the concept of ‘protection motivation’, suggesting that fear-inducing messages can effectively encourage individuals to take preventive action against potential harm. The theory posits that four key beliefs enhance an individual’s intention to protect themselves. These beliefs include the perceived seriousness of the threat, the belief in one’s likelihood of experiencing the threat, confidence in the effectiveness of the protective behaviour and the belief in one’s capability to successfully perform the recommended behaviour. Conversely, PMT also states that perceived costs associated with adopting risk-reduction behaviours and perceived benefits of engaging in risk-enhancing behaviours can weaken protective intentions. In the context of unintentional childhood injuries, PMT suggests that information about the potential consequences of such injuries, like falls or poisoning, could heighten fear and amplify mothers’ perceptions of the severity and likelihood of these injuries affecting their children. If mothers feel confident in their ability to prevent these injuries and believe that changing their protective behaviours will lead to positive outcomes, they are likely to express a stronger intention to adopt more protective behaviours.

## Methodology

### Research design

A descriptive cross-sectional survey research design was employed in this study to establish a baseline understanding of the nature of unintentional injuries and the knowledge and attitudes of mothers with children under the age of five. This design is appropriate as a foundational step towards developing targeted primary and secondary prevention programmes.

### Study population

The target population for this study comprised all mothers of children under five attending two infant welfare hospitals in Ogbomoso, Oyo State, Nigeria, from November 2023 to December 2023. Hospital records indicate that approximately 600 children with unintentional injuries received care in the General outpatient Department, Accident and Emergency unit between 01 January 2023 and 30 September 2023.

### Study setting

The research was conducted at two teaching hospitals in Ogbomoso: Bowen University Teaching Hospital (BUTH) and LAUTECH Teaching Hospital. These institutions serve as referral centres, providing comprehensive health care services including family medicine, diagnostic imaging, cancer care, emergency services, dialysis, physiotherapy, nursing education, rehabilitation and paediatric intensive care.

### Sample size and sampling technique

A purposive, convenient sampling technique was employed to select two teaching hospitals as referral centres for unintentional childhood injuries (UCI) within the locality. This approach, consistent with methodological recommendations by Patton (2015) for targeted research in health care settings, ensured that the sample included mothers directly affected by such injuries. A total of 200 mothers of children under five participated in the study, providing a representative sample for analysing knowledge and attitudes towards unintentional childhood injuries. The sample size of 200 was determined based on several factors, including the prevalence of unintentional childhood injuries in the target population, resource availability and the need for statistical power (Peden et al. 2008). Hospital records indicated that approximately 600 children with unintentional injuries were treated between January and September 2023, informing estimates of likely participant numbers. This sample size was chosen to ensure sufficient power for detecting significant differences and associations while being practical given the available time and resources for data collection and analysis. Ultimately, it achieved a balance between statistical relevance and manageability and was calculated to maintain an acceptable margin of error, ensuring the reliability of the survey results (Dillman et al. 2014).

### Instruments for data collection

Researchers developed the Knowledge of Unintentional Childhood Injuries Scale (KUCI) based on a review of relevant literature, including guidelines from the CDC (2012) and the National Center for Injury Prevention and Control’s Action Plan for Child Injury Prevention. This scale was designed to gather information on mothers’ knowledge regarding unintentional childhood injuries. Respondents were provided with three response options: ‘Yes’, ‘No’ and ‘I do not know’. In addition, the Attitude Towards Unintentional Childhood Injuries (ATUCI) Questionnaire was employed to assess mothers’ attitudes. Responses were scored on a 4-point modified Likert scale, with the following options: Strongly Agree (SA), Agree (A), Disagree (D) and Strongly Disagree (SD). Points were allocated as follows: SA = 4, A = 3, D = 2 and SD = 1. The initial scale was tested for validity and reliability, with coefficients ranging from *r* = 0.75 (*p* < 0.001) for the KUCI and r = 0.80 (*p* < 0.001) for the ATUCI. In terms of validity, factor analysis, which aims to test the dimensionality of the scale, revealed that the KUCI and ATUCI demonstrated strong construct validity.

### Procedure for data collection

To ensure data accuracy, the questionnaire was administered by the researchers along with four trained assistants who were health attendants familiar with the study. Prior to data collection, appropriate permissions were obtained from relevant authorities, including heads of units, supervising doctors, nurses and health attendants. The data collection process began with a briefing session for the research team and assistants to review the study objectives, ethical considerations and the administration of the questionnaires. This ensured that all team members were well prepared to address participants’ questions and provide clear instructions.

The questionnaires were distributed in a private setting to maintain participant confidentiality and encourage honest responses. Each participant received a brief explanation of the study’s purpose, the nature of the questions and their right to withdraw at any time. To facilitate a high response rate, the questionnaires were self-administered, and research assistants were available to assist participants with any difficulties they encountered while completing the forms. After completion, the questionnaires were collected immediately to ensure a high retrieval rate. The researchers monitored the collection process closely to address any potential issues and to verify that all ethical protocols were followed throughout the data collection process.

### Ethical considerations

The study adhered strictly to ethical guidelines for research involving human subjects, as outlined in the Declaration of Helsinki, Ethical approval to conduct this study was obtained from Lautech Teaching Hospital and Lautech Teaching Hospital Research Ethics Committee on 29 October 2023 (No. 12-07/2023), ensuring the protection of participants’ rights, confidentiality and well-being throughout the research process. Informed consent was secured from all participants, with both written and oral consent obtained prior to their involvement in the study. This comprehensive approach ensured that participants were fully aware of the study’s purpose, procedures, potential risks and their right to withdraw at any time without any repercussions.

### Data analysis

Data analysis was conducted using frequency counts, simple percentages, pie charts, means, standard deviations and chi-square tests (Fisher’s exact test) to assess associations between variables. These statistical methods facilitated a comprehensive understanding of the knowledge and attitudes of mothers regarding unintentional childhood injuries.

## Results

### Participants’ demographic descriptions

This demographic overview highlights the characteristics of mothers with children under five, providing insights into their age, parity, religious affiliation and educational background. Table 1 shows that many respondents (50%) were aged between 30 years and 39 years, indicating a trend towards older mothers in this demographic. Among the participants, 33% were primiparas (first-time mothers), while 67% were multiparas (those with more than one child). In addition, a significant portion of the mothers (81%) were identified as Christians. Notably, the majority (59%) had not attained formal education.

**TABLE 1:** Participants’ demographic descriptions.

Demographic variable	Frequency	%
**Age (years)**		
Less than 20	3	1.5
20–29	10	5.0
30–39	100	50.0
40 and above	87	43.5
**Total**	**200**	**100.0**
**Parity**		
Primipara	66	33.0
Multipara	134	67.0
**Total**	**200**	**100.0**
**Religion**		
Christianity	162	81.0
Islam	30	15.0
Traditional	8	4.0
**Total**	**200**	**100.0**
**Level of education**		
Primary school	32	16.0
Secondary school	50	25.0
No formal education	118	59.0
**Total**	**200**	**100.0**

### Common types of unintentional injuries among under-five children in Ogbomoso, Oyo State

**FIGURE 1:**
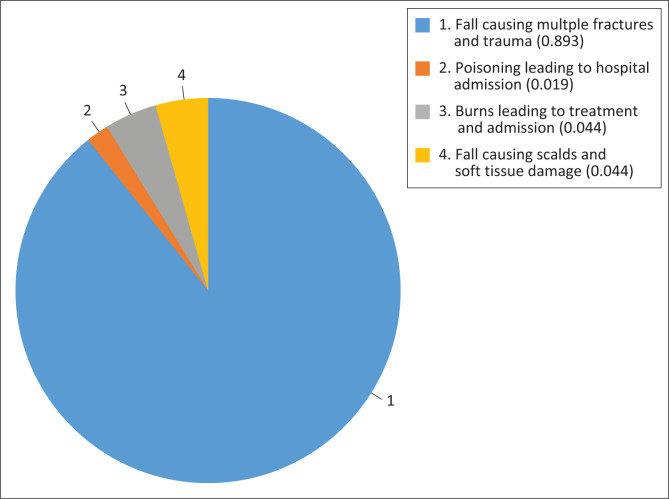
Common types of unintentional injuries among under-five children.

Figure 1 shows that falls, particularly those causing multiple fractures and trauma, are the most common unintentional injuries among under-five children in Oyo State, with 110 respondents (55.0%) reporting such incidents. In addition, 40 respondents (20.0%) reported cases of burns requiring treatment and hospitalisation, while another 40 respondents (20.0%) noted severe falls leading to scalds and soft tissue damage.

### Maternal levels of knowledge about unintentional childhood injuries

As shown in Table 2, the overall weighted mean value of 1.614 falls below the criterion mean of 2.0, indicating a low level of knowledge regarding unintentional childhood injuries among this demographic. The results reveal trends in the knowledge of unintentional childhood injuries among mothers of children under five in Ogbomoso, Oyo State. A significant number of respondents, 99 (45.0%), were unaware that unintentional childhood injuries are preventable. Furthermore, 116 (58.0%) respondents did not recognise common examples of these injuries, such as falls, poisoning, burns and drowning. More than half of the mothers (55.0%) disagreed that a lack of parental supervision is a major risk factor contributing to childhood injuries. In addition, a troubling 70.0% of respondents were not aware that childhood injuries can sometimes be fatal. About 80.0% of mothers did not believe that medications should be kept out of children’s reach, highlighting a critical gap in safety awareness.

**TABLE 2:** Maternal levels of knowledge about unintentional childhood injuries.

Items	Yes		No		I do not know		s.d.
	*n*	%	*n*	%	*n*	%	
Unintentional childhood injuries are preventable	5	2.5	99	49.5	96	48.0	0.55
Examples of unintentional childhood injuries include falls, poisoning, burns and drowning	2	1.0	116	58.0	82	41.0	0.51
Lack of parental supervision is a major risk factor in childhood injuries	7	3.5	110	55.0	83	41.4	0.55
Allowing a child into the kitchen unsupervised is a risk for burns injury among children	5	2.5	123	-	72	36.0	0.52
Unrestricted access to sources of hot water can predispose a child to burn injuries	4	2.0	115	57.5	81	40.5	0.53
During the crawling stage of development, the house must be organised to limit injuries	4	2.0	120	60.0	76	38.0	0.52
Crawling children should be prevented from entering kitchens and bathrooms	5	2.5	99	49.5	96	48.0	0.55
Children should not attempt to make beverages or food with hot water	2	1.0	116	58.0	82	41.0	0.51
Children should be supervised when climbing stairs	7	3.5	110	55.0	83	41.5	0.55
Wet floors contribute to higher incidences of childhood injury	5	2.5	123	61.5	72	36.0	0.52
Children should be properly supervised while playing	4	2.0	115	57.5	81	40.5	0.53
Children should not be left alone with other minors	4	2.0	120	60.0	76	38.0	0.52
All drugs should be kept out of children’s reach	14	7.0	159	79.5	27	13.5	0.55
Falls can lead to permanent disability in some children	13	6.5	68	34.0	119	59.5	0.80
Childhood injuries can be fatal and may lead to death	18	9.0	42	21.0	140	70.0	0.75
Household medicines are a common source of poisoning in children	4	2.0	120	60.0	76	38.0	0.52

Note: Criterion mean: 2.0; Weighted mean: 1.614.

s.d., standard deviation.

### Maternal attitudes towards unintentional childhood injuries

The analysis of maternal attitudes towards unintentional childhood injuries reveals troubling trends among mothers of children under five in Ogbomoso, Oyo State. The data in Table 3 indicate a strong belief in divine protection, with over half of the respondents (59.5%) agreeing that only God can safeguard children, which leads to a diminished focus on injury prevention. In addition, more than two-thirds (69.5%) believe that no amount of supervision can prevent injuries, reflecting a fatalistic view of child safety. Furthermore, 70% of mothers reported that they pray for their children’s safety each morning, suggesting a reliance on spiritual intervention over proactive measures. The overall weighted mean of 1.399, falling below the criterion mean of 2.5, indicates a negative attitude towards preventing unintentional childhood injuries.

**TABLE 3:** Maternal attitudes towards unintentional childhood injuries.

Items	SA	A	D	SD	Mean	s.d.
I can keep any household materials anywhere	162	32	3	3	1.23	0.54
My parents care less about injuries, and I adopt their parenting style	159	27	9	5	1.30	0.67
It is only God that can protect little children; thus, I care less about injury prevention	119	68	6	7	1.50	0.72
No amount of supervision and carefulness can prevent unintentional childhood injuries	139	42	9	10	1.45	0.80
I pray for my children every morning so no matter how careless they are, they cannot be hurt	140	42	11	7	1.42	0.75
Getting injuries is part of growing up of a child	131	54	8	7	1.45	0.73
No child can be without injury, so it is a waste of time trying to protect them	131	56	7	6	1.44	0.70
It’s normal to have naked electric wires in my house	144	42	10	4	1.37	0.67
I can leave my child for a while on the bed without supervision and nothing will happen	143	47	6	4	1.35	0.64
There is nothing wrong if I do not label my bottles	117	76	5	2	1.46	0.60
Pre-school children can sit in the front seat of a car with little or no supervision	76	117	5	2	1.46	0.60

Note: Criterion mean: 2.5; Weighted mean: 1.399.

SA, strongly agree; A, agree; D, disagree; SD, strongly disagree; s.d., standard deviation.

### Association between knowledge scores and socio-demographic characteristics

The association between respondents’ knowledge scores regarding unintentional childhood injuries and their socio-demographic characteristics is summarised in Table 4. The analysis indicates that significant associations were found between knowledge scores and age, religion and level of education (*p* ≤ 0.05).

**TABLE 4:** Association between knowledge scores and socio-demographic characteristics.

Socio-demographic characteristics	Poor knowledge		Fair knowledge		Good knowledge		Chi-square (*χ*^2^)	*df*	*p*-value
	*n*	%	*n*	%	*n*	%			
**Age (years)**	-	-	-	-	-	-	10.38	7	0.001
< 20	6	3.0	4	2.0	3	1.5	-	-	-
30–39	35	17.5	43	21.5	22	11.0	-	-	-
≥ 40	25	33.0	45	22.5	17	8.5	-	-	-
**Parity**	-	-	-	-	-	-	4.03	2	0.501
Primipara	56	28.0	42	21.0	8	4.0	-	-	-
Multipara	40	20.0	29	14.5	25	12.5	-	-	-
**Religion**	-	-	-	-	-	-	15.12	4	0.001
Christianity	60	30.0	52	26.0	50	25.0	-	-	-
Islam	12	6.0	10	5.0	8	4.0	-	-	-
Traditional	3	1.5	3	1.5	2	1.0	-	-	-
**Level of education**	-	-	-	-	-	-	12.17	6	0.002
Primary education	19	9.5	9	4.5	4	2.0	-	-	-
Secondary	20	10.0	17	8.5	13	6.5	-	-	-
No formal education	70	35.0	29	14.5	19	9.5	-	-	-

Note: Fisher’s exact test was used.

## Discussion

The findings of this study, analysed through the PMT framework, provide critical insights into maternal knowledge, attitudes and behaviours concerning unintentional childhood injuries. This discussion integrates our findings with current literature and theoretical perspectives to highlight key themes and intervention strategies. Our study identified falls resulting in fractures and trauma as the most common unintentional injuries, accounting for 55% of cases, followed by burns and scalds with soft tissue damage, each representing 20%. This distribution aligns with global epidemiological data from the WHO Global Injury Report of 2024 and reflects developmental vulnerabilities and environmental risk factors prevalent in low- and middle-income countries, as highlighted by Kumar et al. (2019), as well as Kopits and Cropper (2005). Several factors contribute to the high prevalence of falls, including the developmental stages of young children, hazardous residential environments, limited awareness of preventive measures and inadequate supervision, as documented by Thompson et al. (2013), Adeniran et al. (2008), Rahman, Andersson and Svanström (1998), Abd El-Aty et al. (2005), and Chen et al. (2005).

Despite the high incidence of falls, only 40.5% of mothers recognised that falls could lead to permanent disabilities. This gap in risk assessment suggests a misalignment between actual threats and perceived severity, a pattern also observed in studies conducted in other developing nations by Rahman et al. (2023) and Xiang et al. (2019); addressing this misperception is essential for improving protective behaviours.

A significant association was found between maternal knowledge scores and socio-demographic characteristics, with a statistical significance of *p* ≤ 0.05. This finding supports research by Ibrahim et al. (2024) and Ahmed et al. (2022), which indicates that maternal education significantly influences injury prevention awareness and practices. Educated mothers demonstrated higher awareness of injury risks, with 72% showing understanding compared to 28% among those with lower educational attainment. Formal education also correlated with better preventive behaviours, with a statistical significance of *p* ≤ 0.01, and 65% of educated mothers demonstrated greater proficiency in first aid knowledge compared to 35% among less-educated mothers, aligning with findings by Aktürk (2016). This correlation underscores the need for targeted education programmes to enhance threat awareness and response efficacy.

Religious and cultural beliefs emerged as significant influences on injury prevention attitudes. Most mothers, accounting for 59.5%, prioritised divine protection overactive preventive measures. This finding aligns with research by Okonkwo et al. (2023) and Nduagubam et al. (2022), who documented similar patterns in West African contexts. Within the PMT framework, this perspective impacts response efficacy, as mothers may perceive spiritual protection as more effective than tangible preventive measures. Culturally sensitive interventions are needed to integrate traditional beliefs with evidence-based safety practices.

Burns and scalds accounted for 20% of reported injuries, presenting severe long-term health risks. Alarmingly, 80% of mothers demonstrated poor knowledge regarding burn prevention, particularly the importance of keeping hazardous materials out of children’s reach. This finding aligns with research by Farzan et al. (2023) and El-Sabely, Yassin and Zaher (2014), which reported similar knowledge gaps in developing countries. The combination of limited awareness and unsafe household practices, as also noted by Riyadh et al. (2013), underscores the need for targeted burn prevention programmes that emphasise both risk identification and behavioural change.

Applying PMT to our findings highlights critical aspects of maternal risk perception and protective behaviours. Regarding threat appraisal, only 30% of mothers recognised the potential severity of injuries, and 45% acknowledged their children’s susceptibility to harm, consistent with findings by Lee, Walia and Forbes (2012). Concerning coping appraisal, response efficacy remained low, with only 40% believing in the effectiveness of preventive measures, while self-efficacy was even lower at 35%, indicating limited confidence in injury prevention strategies, as similarly reported by Isaac et al. (2022).

Given these insights, a multi-faceted intervention approach is necessary. Educational programmes should focus on structured knowledge enhancement initiatives that emphasise injury prevention strategies, culturally adapted training materials, and hands-on skills development, as recommended by Rabiu and Ogundipe (2022). Community-based support should involve the formation of local support networks for mothers, collaborative safety initiatives and active engagement of community health workers. Policy recommendations should include strengthened safety regulations, improved healthcare accessibility, and the integration of mandatory safety education in maternal and child health programmes, as suggested by Samah (2022).

## Clinical implications

Findings from this study suggest several key areas for clinical intervention. Health care provider training should be enhanced to include assessment of household safety risks, culturally competent counselling and integration of religious perspectives in safety education. Preventive care programmes should incorporate routine safety assessments during well-child visits, targeted interventions for high-risk groups and expanded community outreach programmes. Resource development should focus on the creation of culturally appropriate safety education materials, multilingual safety guides and visual aids to enhance injury prevention awareness. Based on our findings, several recommendations are proposed. Educational initiatives should prioritise the development of comprehensive safety education programmes, integration of cultural and religious perspectives and ongoing evaluation of intervention effectiveness. Health care improvements should focus on enhanced injury surveillance systems, improved emergency response protocols and better documentation of injury patterns. Community engagement should involve the establishment of mother support groups, local safety awareness campaigns and collaborative community-based prevention programmes.

## Conclusion

This study highlights falls, burns and scalds as the predominant unintentional injuries among under-five children in Ogbomoso, Nigeria. Using the PMT framework, critical gaps in both threat awareness and coping strategies among mothers were identified. The significant associations between maternal education, knowledge and attitudes towards injury prevention underscore the need for targeted interventions addressing both cognitive and behavioural aspects of injury prevention. Future efforts should focus on developing structured education programmes tailored to different sociocultural contexts, integrating religious beliefs with evidence-based prevention strategies and fostering community support systems. A combination of educational, clinical and policy-driven interventions is essential to enhance maternal protective behaviours and reduce the burden of childhood injuries in Nigeria and similar settings.
